# Supplementary material for: Replication Protein A, the Main Eukaryotic Single-Stranded DNA Binding Protein, a Focal Point in Cellular DNA Metabolism
Source: Int J Mol Sci. 2024 Jan 2;25(1):588. doi: 10.3390/ijms25010588 (PMC10779431; doi:10.3390/ijms25010588)
Supplement: Supplementary file 1 [file ijms-25-00588-s001.zip › ijms-2776184-supplementary.pdf]

Supplemental Information:

A

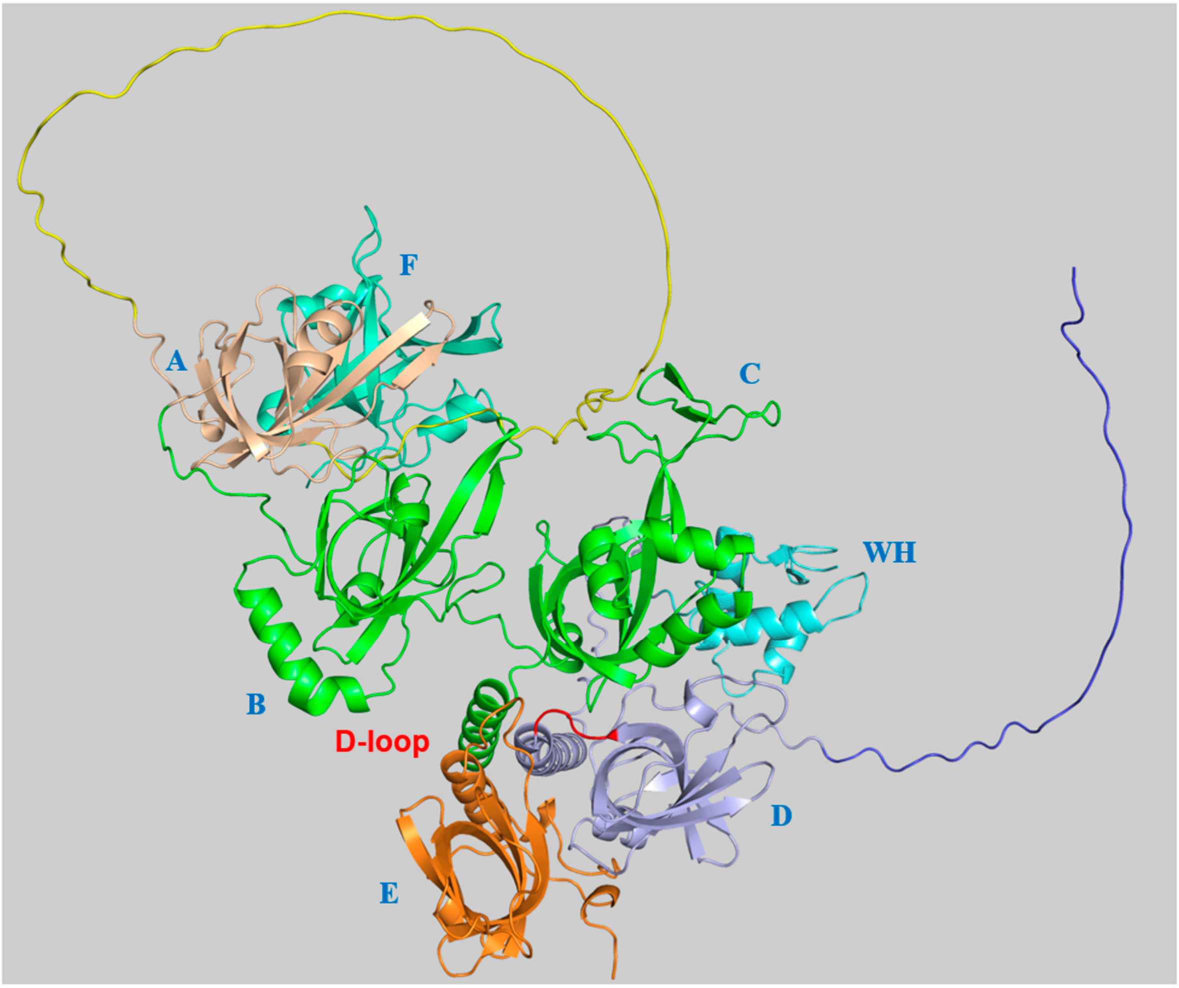

B

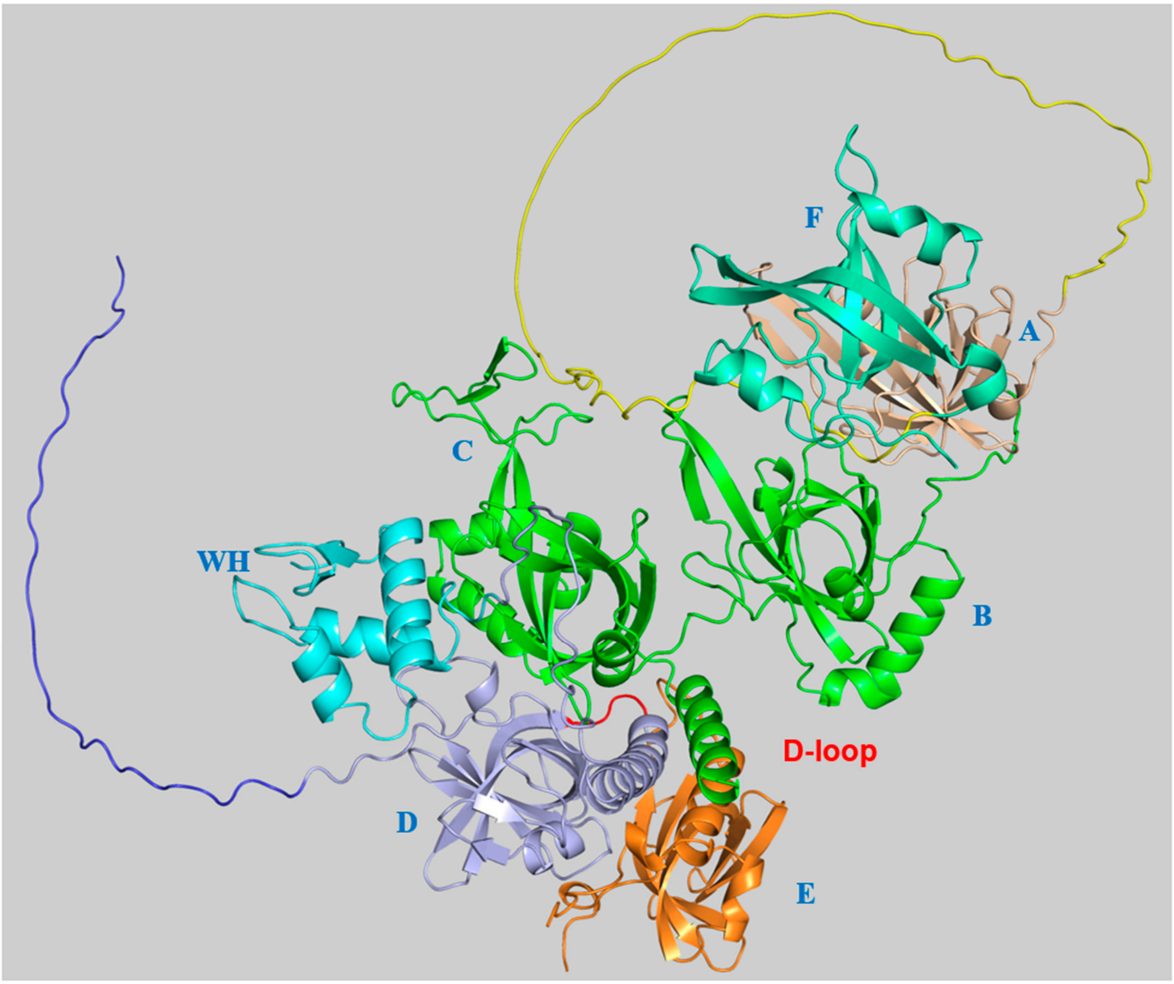

Supplemental Figure S1. The Predicted Structure of Human RPA. Panels **A** and **B** present images of the RPA trimer predicted by AlphaFold [25] and visualized using the PyMOL 2.5.5 program (Schroedinger Inc. (USA)). The light green and light brown colours represent the DNA binding domains of RPA70 DBD-F and DBD-A, respectively. The linker between these two domains is shown in yellow. DBD-B, DBD-C and the short linker between the two domains are presented in green. The RPA32 phoshorylation sequence is in dark blue [23], whereas WH domain of RPA32 is in cyan. DBD-D and the aa connecting DBD-D and WH are shown in light blue. In DBD-D the loop containing the conserved aa D151 is called D loop (see also figure 1) and shown in red. It is important to note that the linker regions of RPA70 and RPA32 plus the N-terminal phosphorylation sequences of RPA32 are predicted with a very low per-residue model confidence score (pLDDT) suggesting they are unstructured without ligands or modifications. Panel **B** is the same predicted AlphaFold structure as in **A**, but with its atomic coordinates of atoms being rotated (arguments of rotation: y-axis and 180 degrees) using PyMol. The predicted structure suggests that DBD-F (light green) and WH domain (cyan), which are both important for RPA-protein interactions, are on the same site of the RPA molecule. It is important to note that the linker regions of RPA70 and RPA32 plus the N-terminal phosphorylation sequences of RPA32 have a very low per-residue model confidence score (pLDDT) and might be unstructured. Due to very low pDDDT scores and the N-linker sequence of RPA70 other arrangements for DBD-F within the RPA complex are also possible. However, published, experimental RPA structures including the *Ustilago maydis* RPA structure (PDB 4gop [13] see also figure 1, panels B and C) have not yet included DBD-F, the N-terminal linker sequence of RPA70, the WH domain, and the N-terminus of RPA32 in a structure of the heterotrimeric RPA complex. Therefore, the presented structure is very helpful for further discussion of RPA functions. The RPA domains are marked with blue letters, A to F (DBD-A to F) and WH.

```
hSTN1     ------------------------------MQPGSSR-CEEETPSLLWGLDPVFLAFAKL   29
hRPA32    MWNSGFESYGSS---SYGGAGGYTQSPGGFGSPAP-SQAEKKSRARA-------QHIVPC   49
uRPA32    MFNNGFGGESNPYAQNYSTGGG--ASGGGFLANGSQNDSPSGKKAGN-------NTLRPV   51
                                           .    . . . :           :

hSTN1     YIRDILDMKESRQVPGVFLYNGHPIKQVDVLGTVIGVRERDAFYSYGVDDST-GVINCIC   88
hRPA32    TISQLLSA-T--LVDEVFRIGNVEISQVTIVGIIRHAEKAPTNIVYKIDDMTAAPMDVRQ   106
uRPA32    TIRQILNA-EQPHPDAEFILDGAELGQLTFVAVVRNISRNATNVAYSVEDGT-GQIEVRQ   109
           * ::*.          *  ..  : *: .:. :    .  :   * ::* * . ::

hSTN1     WKKLNTESVSAAPSAARELSLTSQLKKLQETIEQKTKIEIGDTIRVRGSIRTYREEREIH   148
hRPA32    WVDTDDT-SSEN-----------------------TVVPPETYVKVAGHLRSFQNKKSLV   142
uRPA32    WLDSSSDDSSKA-----------------------SEIRNNVYVRVLGTLKSFQNRRSIS   146
          * . .    *                         : :     ::* * ::::::.:.:

hSTN1     ATTYYKVDDPVWNIQIARMLELPT---IYRKVYDQPFHSSALEKEEALSNPGALDLPSLT   205
hRPA32    AFKIMPLEDM--NEFTTHILEVINAHMVLSKANSQPSAGRAPISNPGMSEAGNFGGNSFM   200
uRPA32    SGHMRPVIDY--NEVMFHRLEAVHAHLQATRGPTASAL-KGATSTALFNQAGGLESNDMN   203
          :     : *   *    : **         :         .  .   :.: * :   .:

hSTN1     SLLSEKAKEFLMENRVQSFYQQELEMVESLLSLANQPVIHSASSDQVNFKK--DTTSKAI   263
hRPA32    PANG--------------LT----VAQNQVLNL--IKA--CPRPEGLNFQDLKNQLKHMS   238
uRPA32    AYSGSKK------QDVNDLYKNLGELPQQIMSIVTRES--QNHPDGVHVSLIARLLNGVD   255
             .              :        :.::.:           : ::..      .

hSTN1     HSIFKNAIQLLQEKGLVFQKDDGFDNLYYVTREDKDLHRKIHRIIQQDCQKPNHMEKGCH   323
hRPA32    VSSIKQAVDFLSNEGHIYSTVDD-----------DHFKSTD-------------AE----   270
uRPA32    VNDVKSAVEDLSSDGYLYTAADD-----------NHVLPTA-------------------   285
           . .*.*:: *...* ::   *.           ...  .

hSTN1     FLHILACARLSIRPGLSEAVLQQVLELLEDQSDIVSTMEHYYTAF   368
hRPA32    ---------------------------------------------   270
uRPA32    ---------------------------------------------   285
```

Supplemental Figure S2. The multiple sequence alignment of human STN1, human and *Ustilago maydis* RPA32 (hSTN1, hRPA32 and uRPa32, respectively) shows that the aspartates D157 of STN1, D151 of human RPA32, and D155 of *Ustilago maydis* RPA32 are conserved (red D in bold and underlined). The loop aa of the three proteins (see also aligned protein structures in Figure 1c and 1E) are shown in red. The OB-fold sequences of the proteins are presented in bold. The underlined sequence of hSTN1 was omitted in the structure alignments of Figure 1C and 1E.
